# Clinical Perspectives on the Use of Computer Vision in Glaucoma Screening

**DOI:** 10.3390/medicina60030428

**Published:** 2024-03-02

**Authors:** José Camara, Antonio Cunha

**Affiliations:** 1 Engineering Department, Universidade Tras-os-Montes (UTAD), 5000801 Vila Real, Portugal; 2 INESCTEC—INESC Technology and Science, 4200465 Porto, Portugal

**Keywords:** early manifest glaucoma trial, pre-perimetric glaucoma screening, glaucoma screening cost, glaucoma screening by photos, glaucoma screening by teleophthalmology

## Abstract

Glaucoma is one of the leading causes of irreversible blindness in the world. Early diagnosis and treatment increase the chances of preserving vision. However, despite advances in techniques for the functional and structural assessment of the retina, specialists still encounter many challenges, in part due to the different presentations of the standard optic nerve head (ONH) in the population, the lack of explicit references that define the limits of glaucomatous optic neuropathy (GON), specialist experience, and the quality of patients’ responses to some ancillary exams. Computer vision uses deep learning (DL) methodologies, successfully applied to assist in the diagnosis and progression of GON, with the potential to provide objective references for classification, avoiding possible biases in experts’ decisions. To this end, studies have used color fundus photographs (CFPs), functional exams such as visual field (VF), and structural exams such as optical coherence tomography (OCT). However, it is still necessary to know the minimum limits of detection of GON characteristics performed through these methodologies. This study analyzes the use of deep learning (DL) methodologies in the various stages of glaucoma screening compared to the clinic to reduce the costs of GON assessment and the work carried out by specialists, to improve the speed of diagnosis, and to homogenize opinions. It concludes that the DL methodologies used in automated glaucoma screening can bring more robust results closer to reality.

## 1. Introduction

With the growth and aging of the population, the demand for ophthalmological services for age-related diseases such as glaucoma, cataracts, diabetic eye disease, and macular degeneration increases and brings increasing logistical difficulties in meeting these demands, especially in locations far from large centers. On the other hand, it drives the development and improvement of new techniques for the population screening of these diseases.

Glaucoma is one of the leading causes of irreversible blindness globally [1]. It is estimated that at least 2.2 billion people worldwide have a visual impairment, of which at least one billion have a visual impairment that could have been prevented or has not yet been addressed [2]. Medical evidence shows that early diagnosis and treatment in the early stages of glaucoma significantly reduce costs by delaying partial or total disability resulting from blindness, such as the direct costs related to medical fees, medication costs, and the indirect costs related to days not worked, caregiver fees, and rehabilitation.

In this aspect, using tools such as artificial intelligence (AI) can assist medical specialists in the growing population demand.

To obtain a clinical sense, the specialists use retinal image analysis with clinical data and complementary exams such as VF and OCT. Still, it is a process that is often time-consuming, dependent on the experience of professionals acquired over a long period of clinical and personal experience, and dependent on emotional and psychological factors. Furthermore, studies show low agreement in optic nerve assessment and only moderate inter-examiner reproducibility for detecting glaucomatous features [3].

Computer vision is a field of AI that studies algorithms that are capable of visually interpreting information obtained in digital images, noticing defects imperceptible to human capabilities. Automated GON screening using deep learning architectures involving the acquisition of images, including by portable means, which can be stored and distributed to ophthalmological centers, together with patient records, through teleophthalmology, which lower costs compared to in-person examination, helps with diagnosis, and reduces waiting lists for ophthalmological care.

DL techniques applied at various stages of screening demonstrate the potential to screen GON and other retinal diseases from images quickly and accurately with efficacy compared to expert standards [4,5,6], facilitating the work of professionals who seek clinical meaning in the set of data obtained from the patient, but, to date, a single algorithm has not been validated to predict the incidence and progression of glaucoma [7]. Furthermore, OCT and VF have been used as truth sets to validate CFPs, avoiding bias in experts’ decisions [4].

The importance of this study is to analyze the use of DL in glaucoma screening from a clinical perspective, proposing more efficient and lower-cost solutions that are less dependent on specialist interpretation bias.

### 1.1. Research Objective and Main Contributions

Recognition of the glaucomatous papilla is complex, especially in its early forms, due to the anatomical variability of the optic nerve in the population and the lack of universally accepted parameters. In medical practice, multimodal data are used, i.e., originating from different stages of the clinical examination, including images and complementary, structural, and physiological exams, made available to specialists, increasing the possibility of correct diagnoses in many cases, and improving the performance of AI analysis. Furthermore, interpreting data and images transmitted over the internet using telemedicine techniques assists population demands and the specialists’ work, which automated AI techniques have improved.

The objectives of this work were as follows: (1) To correlate the use of multimodal data used in clinical practice to offer better solutions in AI-automated methods for glaucomatous papilla recognition. For this purpose, some medical concepts were described to explain the relevance of using multimodal data in clinical practice with potential application in DL methodologies. (2) To identify research that used data from more than one source to classify glaucoma using DL technologies and identify the main challenges.

Some questions were proposed to obtain a better understanding of the main scenarios of glaucoma diagnosis.

Q1. What are the main challenges in the clinical recognition of a glaucomatous optic nerve, especially in its early stages?

Q2. What literary references defined the diagnostic limits of AI glaucomatous optic neuropathy using multimodal data?

Q3. What are the main challenges in this area of research?

The first question concerns the complexity of recognizing the glaucomatous papilla in medical practice (summarized below in Section 2.1), considering factors such as the lack of universally accepted biomarkers and the anatomical diversity of the optic nerve head in the population.

The second question is defined in the state of the art (summarized below in Section 3.2) and brought literary references that used data to analyze the glaucomatous papilla using DL methodologies. We saw that AI has the potential to track glaucoma and interpret the OCT and the CV.

The third question refers to the challenges of recognizing glaucoma using AI and establishing confidence limits. The literature generally mentions that different combinations of multimodal data for glaucomatous optic nerve screening can improve the results of suspected and early cases, facilitating population demands for glaucoma screening and remembering that a qualified ophthalmologist must accompany glaucoma screening.

### 1.2. Materials and Methods

This review aimed to select studies that present a combination of multimodal data to classify the glaucomatous optic nerve. A comprehensive literary review was carried out and selected using inclusion and exclusion criteria of subjects and data selection.

Based on medical practice, the inclusion criteria prioritized selecting studies investigating glaucoma through AI from two or more multimodal data sources. Among them, objective approaches were considered, which do not depend on the emotional and psychological factors or the experience of specialists, such as optical coherence tomography (OCT) examinations, and subjective approaches that depend on personal characteristics, such as the classification made by specialists using color background photographs.

Studies that used only one data source for glaucoma screening and those that did not use AI to classify the disease were excluded.

The keywords “Early Manifest Glaucoma Trial, pre-perimetric glaucoma screening, glaucoma screening cost, glaucoma screening by photos, glaucoma screening by teleophthalmology” were added to the search. The search for publications was carried out mainly through Google Scholar, Scopus, MDPI, Springer, Medline, AAO, and Cochrane.

## 2. Some Approaches to Medical Practice in Glaucoma

In glaucoma screening, experts analyze clinical data from optic nerve head analysis, biomarkers, and ancillary exams based on their experiences [8]. However, studies show low agreement between examiners in evaluating stereo photographs and only moderate reproducibility to detect glaucomatous features in the optic disc [3]. At this point, automated screening can represent an advantage.

Although optic disc cupping is a diagnostic feature of glaucoma, there is an excellent diversity of presentation among healthy individuals. Larger discs tend to have larger cup-to-optic disc vertical diameter ratio (vCDR) measurements. Therefore, although vCDR is a sensitive indicator, it should not be used alone to diagnose glaucoma because it can lead to high false favorable rates [9].

In more distant locations, with limited resources and a high demand for care, glaucoma screening may depend only on a slit-lamp fundus examination, often performed without mydriasis, where the values of the disc area ratio and excavation (CDR) [9] generate a high rate of suspected diagnoses with unnecessary referrals for additional tests, thus compromising patient confidence. In these cases, AI could solve some of these demands using teleophthalmology.

Ophthalmologists overestimate physiologically enlarged cups in larger optic discs or underestimate them in small discs; therefore, models that depend on human gradations to establish the reference standard may present severe limitations in defining the characteristics of the optic nerve.

Table 1 exemplifies some clinical parameters used to screen normal, suspicious, and glaucomatous eyes and the approaches that can be used. In normal eyes, the optic nerve does not present defects in the neural layer nor the subsidiary exams; the suspect has flaws in the neural layer without correspondence with the subsidiary exams.

### 2.1. Clinical Practice in the Analysis of the Optic Papilla

The transparency of the ocular tissue media allows the viewing and documenting of optic nerve head images. However, the diagnosis of glaucoma should be made more than just through pictures but through available clinical data and complementary physiological and structural exams, as previously mentioned.

The signs of glaucomatous optic neuropathy (GON) are based on an increase in the cup/disc ratio, loss of the ISNT pattern, changes in the path of the vessels, presence of the lamina cribrosa, and hemorrhages. The diagnosis of certainty is the correspondence of the fiber layer defect in at least two visual field tests. Recent discoveries suggest that Bruch’s membrane opening (BMO), defined using spectral domain optical coherence tomography (sdOCT) at the disc margins, can serve as a reference for the diagnosis and progression of GON [10].

Table 2 shows that many characteristics of standard images are also present in GON; in addition, there is a lack of consensus to define the references between normal and glaucomatous eyes and reinforces the importance of evaluating not only the images but the entire set of data and examines additional information for a more accurate diagnosis.

### 2.2. Visual Field (VF) in Glaucoma

Campimetry or automated perimetry (white on white) is considered the gold standard for evaluating the visual field in glaucoma [11], as it reflects the loss of fibers secondary to glaucomatous disease progression.

The ophthalmologist interprets the automated perimetry by combining risk factors, appearance of the optic nerve, age, ocular pressure (IOP), and family history of glaucoma [12].

Visual field tests require good patient cooperation. Changes in the first exams may present some inconsistency due to failures related to fatigue, patient learning, uncorrected refractive errors, high rate of false-positive responses, inadequate occlusion of the contralateral eye, weak lamp, and misadjustments of the device itself. They may suffer considerable test–retest variability [13,14], especially in people with advanced VF damage [15].

Visual field defects in glaucoma must be reproducible and correspond to the established glaucomatous damage. An increase can be seen in the progression of the perimetric weakness in the number of scotomas, enlargement of their areas, increase in depth, and deterioration of global indices [13].

Most studies using DL use image classification methodologies to screen and study the progression of glaucoma, and many diagnoses can be anticipated by applying DL technologies integrated with segmentation, regression, localization, and generative model techniques to predict future VFs with spatial information. A VF embedded with clinical data such as intraocular pressure (IOP), medication, and medical history assists clinical decision-making and allows personalized treatment for each patient [16].

### 2.3. Optical Coherence Tomography (OCT)

OCT is a non-contact optical imaging technique that uses low coherence interferometry to measure the light reflected from different layers of the retina and optic nerve, providing quantitative measurements of the structures of the posterior segment of the eye, notably the macula, limits of the head of the optic nerve, and excavation, comparing them to a normative database to assist the ophthalmologist in the diagnosis and follow-up of retinal diseases.

Its use for diagnosing glaucoma is indicated in eyes suspected of pre-perimetric glaucoma. Still, it does not replace the need to use retinography for the structural documentation of the optic nerve for both the diagnosis and assessment of progression, i.e., a positive or negative result obtained by OCT alone does not confirm or exclude the diagnosis and progression of glaucoma [15]. The evaluation should include the patient’s medical history, detailed ophthalmological examination, OCT optic disc parameters, and visual field tests [17].

For the detection of pre-perimetric glaucoma, the recommendation is to integrate OCT data with other clinical information to detect glaucomatous damage in patients without an apparent campimetric defect [18,19], seeking a clinical sense. Structural damage assessment involves numerous topographic parameters related to the ONH and macula and is performed imperfectly by the machine’s software, with errors of the scans between 19.9% and 46.3% [20]. Thus, the assessment of the ONH using color fundus photos is a more attractive option than OCT in terms of global screening, considering the portability and lower costs of acquiring color fundus photos [21].

Using sdOCT further improved axial resolution, scanning speed, and diagnostic accuracy compared to previous OCT technologies [22]. However, human experience in interpreting sdOCT still needs to be improved; sdOCT technology is expensive and not easily portable, limiting the feasibility of its widespread adoption in screening efforts. Recent findings suggest that Bruch’s membrane opening (BMO), defined using sdOCT at the disc margins, may be a reference for diagnosing and progressing GON [10].

Deep learning (DL) technology associated with OCT demonstrates efficiency, precision, and exemplary performance in interpreting the exam and discriminating glaucomatous eyes from normal eyes [22,23], contributing to improving diagnosis and reducing the precious time and involvement of professional experts [24].

Table 3 summarizes the stages of glaucoma screening in terms of costs, portability, and diagnostic interpretation, highlighting that image interpretation is a cheap method, still used in detection through sequential analysis, and admitting the use of portable devices to obtain and transmit images via telemedicine. On the other hand, it presents disadvantages such as the variable quality of the photos obtained by different devices, the lack of universally accepted references to characterize normal from glaucomatous ONH, and the dependence on the examiner’s experience.

### 2.4. Transmission and Storage of Data through Teleophthalmology

Teleophthalmology uses technologies to provide remote eye care that have evolved rapidly with smartphones, fifth generation wireless communication, and artificial intelligence [25].

It is based on the storage and forwarding of eye images in real time to screening centers [26]; it can be improved using image processing techniques and artificial intelligence [27], increasing the offer of diagnoses, qualifying and helping to reduce queues’ waiting time for ophthalmological care [28], improving the effectiveness of specialized centers [29] and lowering costs compared to face-to-face examination [30,31].

Data collection, storage, and transmission can combine teleophthalmology with DL techniques in ocular imaging as a potential solution to track, diagnose, and monitor major ocular diseases for patients in primary care and community settings [32]. The data can be used to assess the incidence and progression of the disease in real time [25]. They may represent a viable and effective resource to increase access to care and identify the most common causes of blindness and its risk factors [33]. Ting and colleagues proposed a DL model to screen glaucoma in images transmitted via teleophthalmology. They obtained an ROC curve of 0.942, a sensitivity of 96.4%, and a specificity of 87.2% [34].

In the future, these techniques could play a fundamental role in screening for chronic eye diseases, increasing the accessibility of screening for rural and remote populations.

## 3. Computer Vision and Artificial Intelligence

### 3.1. Concepts

Computer vision is a field of artificial intelligence (AI) that studies algorithms and systems capable of understanding, analyzing, and visually interpreting information obtained from digital images, videos, and other visual inputs, noticing imperceptible defects in human capabilities, being an essential tool in helping diagnosis [8]. In contrast, the experts’ vision is based on experiences acquired over a long period of clinical and personal experience, dependent on physical effort and psychological state.

Convolutional neural networks (CNNs) encompass deep learning (DL) algorithms that use computer vision and image processing. They are designed to recognize patterns and extract features from images through successive layers of representations that analyze the data repeatedly and allow the machine to learn independently without programming [35]. Deep learning (DL) is a subset of artificial intelligence (AI) based on neural networks that use an active learning strategy in the automated detection of glaucoma based on fundus images [36,37]. It can recognize patterns with glaucomatous features in images quickly and accurately [8], achieving a robust performance in detecting other retinal pathologies such as diabetic retinopathy and retinopathy of prematurity, macular edema, and age-related macular degeneration [32], with the potential to assist specialists in mass screening of glaucoma [38], reducing costs, and offering the potential to solve complex problems involving large datasets with medical images and classify diseases with a good innovative perspective for the introduction of individualized medicine and the optimization of diagnosis and therapy, screening, and prognosis [22,39,40], with less dependence on the examiner’s experience [5,41,42], demonstrating the potential for implementation of large-scale screening protocols in the population, to screen for glaucomatous papilla in several evolutionary stages and monitor treatments [43].

Computer-aided automatic screening for glaucoma detection from fundus images is based on the analysis of features such as the optic cup-disk ratio, extracted from segmentation results [44], optic neuroretinal border, and ISNT rules, presenting homogeneous and clinically significant results [38,45]. For this purpose, different strategies are used to segment the disc and optical cup based on the region of interest (ROI). The use of CDRs has shown to have good application in segmentation. The results of the glaucoma assessment using these indicators were good results from the area under curve (AUC) metric (AUC between 0.79 and 0.96) [46]. The CDRs computed using the segmented masks were very close to the ground truth (GT) masks classified by experts, reinforcing that CNNs can make an assessment like that carried out by a clinician. However, in most works, the validation of DL algorithms was compared to human classification results as a reference standard, occurring in the same way with public databases [47], an approach that can present severe limitations, as they tend to exaggerate or underestimate the probability of glaucoma given the significant variability of the optic disc region, the low reproducibility and inter-examiner agreement, the different experiences of the evaluators, and the lack of references to screen for the glaucomatous papilla [4].

The diagnostic accuracy of the model using color photographs suggests that deep learning (DL)-based architectures have the potential to standardize and automate the classification of chronic open-angle glaucoma. However, further studies are needed to implement these technologies [36,37] and bring them closer to reality by analyzing a set of data less dependent on human interpretation, including retinal photographs and clinical markers, symptoms, ocular pressure, family and personal history [47], and complementary exam results such as VF and OCT.

### 3.2. State of the Art

DL methodologies have applications in glaucoma screening by using color images of the optic nerve head, structural (sdOCT), and functional (VF) complementary exams individually or together, demonstrating the potential for diagnosis, prediction of GON progression, and the use of complementary exams as an objective reference for classification, avoiding the bias of subjective interpretation based on the experience of experts [5].

The studies show applications at different stages of glaucoma screening, as follows: fundus photographs, initial VF, and optical coherence tomography for diagnosis. They also propose a standardized reference for validating GON and detecting other retinal pathologies. However, it is essential to remember that a qualified ophthalmologist must diagnose glaucoma using patient data and specific tests to confirm the diagnosis.

Using color fundus photographs (CFPs), Neto [46] analyzed a methodology for glaucoma screening based on algorithms taught with public databases RIM-One r3, DRISHTI-GS, and REFUGE applied in CFPs, referenced by glaucoma experts. It obtained an IoU of 0.81 and a Dice of 0.96, which indicates that the accuracy of the cup segmentation technique is also accurate, with a good overlap with the reference mask.

DL methodologies were applied to the VF in the study by Shuldiner [48], who used DL models on the VF to predict the future progression of glaucoma by training algorithms with reliability metrics based on the initial VF and age and obtained ROC (receiver operating characteristic) with a value of 0.72, indicating a good test performance. The 95% confidence interval (CI) means that there is a 95% probability that the actual AUC value is in the range of 0.70–0.75.

Kucur [49] used a CNN methodology to discriminate between normal eyes and those with early glaucoma through standard and pre-perimetric VFs. The results indicate that the model had an average accuracy of 87.4% for Rotterdam and 96.8% for Budapest.

Huang [50] evaluated visual field loss (VF) in a deep learning (DL) system with results referenced by glaucoma experts and obtained an ROC of 0.93, indicating the model’s high ability to distinguish between true-positives and false-positives, while the accuracy of 85% suggests that the model correctly classified 85% of the cases.

Wen [16] evaluated the VF prediction ability of the 24-2 strategy of DL (deep learning). The 95% confidence interval between 2.45 dB and 2.48 dB indicates that if the experiment were repeated several times, in 95% of cases, the MAE would be within this range. These results suggest a good performance in VF prediction, but further studies are still needed.

Eslami et al. [51] evaluated models to predict VF loss using an independent population. The PMAE 95% confidence intervals were 2.21 to 2.24 dB for CNNs and 2.56 to 2.61 for RNNs, indicating a good performance.

Kihara used DL methodologies in CFPs, VF, and OCT [15], and used two network models to predict VF results from CFPs and OCT. The mean absolute error (PMAE) was 0.485 dB with a 95% confidence interval between 0.438 dB and 0.533 dB compared to the disk image alone and 0.060 dB with a 95% confidence interval between 0.047 dB and 0.073 dB compared to the OCT alone, indicating that using CFPs and OCT in conjunction with DL models can significantly improve the accuracy of VF predictions.

Lim [52] suggests a CNN model to aid the diagnosis of glaucoma using fundus images (CFPs) in eyes with greater axial length (myopia) with an AUROC result of 93.9%, indicating a high detection capacity with a meager error rate.

Table 4 shows studies with DL methodologies applied to color fundus photos (FCF), clinical data (DC), visual field (VF), and optical coherence tomography (OCT). Results referenced with glaucoma specialists (E), incorporation of telemedicine (T), inclusion and exclusion criteria (CE), disease stage classification/VF prediction (R), validation of the results of the method studied by the ROC curve, accuracy (Acc), sensitivity (SE), and specificity (SP)

Mariottoni [4] proposed a reference standard for defining glaucomatous optic neuropathy based on OCT + VF + ONH defects in PFCs with expert-referenced results. It obtained neuropathy (GON) at 99.8%, and ONH at 0.03%, suggesting a good ability to distinguish individuals with and without GON, moderate knowledge in identifying individuals with GON, and a high capacity in identifying individuals without GON. The ONH value of 0.03% suggests that the test cannot identify individuals with optic disc defects.

Noury [54] developed a 3D DL algorithm to detect glaucoma using sdOCT and CFPs in population samples in four countries, divided into normal and glaucomatous eyes, with results referenced by glaucoma experts, obtaining an AUC between 0.91 and 0.99, indicating that the algorithm had a high performance in detecting glaucoma.

Ting [34] analyzed the detection of various retinal pathologies using DL methodologies with results referenced by glaucoma experts. The results indicate good performance in detecting retinal pathologies with AUCs ranging between 0.931 and 0.942.

Singh [24] suggested a tool to evaluate DL models applied in OCT to evaluate glaucoma, using standard and glaucomatous OCT images. Experts referenced the results. It achieved 95.68% accuracy using VGG16.

These studies demonstrate that DL methodologies have the potential to screen glaucoma, interpret OCT and VF, and use them as references for results, thus avoiding the bias of specialist experience, bringing greater security of results, and enabling the diagnosis of early forms, prediction of disease progression based on VF data and differentiation between ocular diseases [34].

New studies will be necessary to compare the results of automated methodologies with those carried out by specialists in different populations and also to study the importance of including the lamina cribriformis obtained using sdOCT as a reference measure in the evolution of excavation and the detection of glaucoma [54].

## 4. Discussion

The present study analyzed how the different stages of computer vision can offer better solutions in glaucoma screening and create more objective references that are less dependent on specialists.

Despite advances in technology, diagnosing glaucoma remains a challenging task and depends on the experience of professionals. The expansion of the use of DL techniques in glaucoma screening processes gives a new impetus towards more accurate results that are less dependent on specialists, bringing automated analysis of the optic papilla closer to the real world; in addition, they can help specialists in the arduous task of triage glaucoma, homogenizing opinions, and producing objective references.

A DL model can recognize disease features in images, VF, and sdOCT and predict glaucoma progression from the VF (see Table 4). However, it requires a large dataset for training. ML algorithms used to predict the risk of rapid glaucoma progression based on initial VF testing [48] can predict the risk of glaucoma onset and advancement based on color fundus photos with an excellent predictive performance (AUROCs 0. 87) [7].

Recent studies show that the convergence of DL methodologies applied at different stages of glaucoma screening increases the speed and quality of results, reducing costs and providing reference standards that are less dependent on the subjectivity of specialist assessment.

The usefulness of using DL techniques must be analyzed from a statistical point of view. As an example, from the work of Ting et al. [34], a DL system was used to screen diabetic retinopathy, glaucoma, and senile macular degeneration with AUC 0.958; 0.942; and 0.931, sensitivity 91.1%; 96.4%; and 93.2% and specificities 91.6%; 87.2%; and 93.2%, respectively. Considering population values, we would have around 12.8% false-positive results in the population; that is, more than 10% of healthy individuals would be diagnosed as “glaucoma suspects” and would be unnecessarily referred for additional tests, in addition to the psychological burden resulting from the suspicion.

VF data have been leveraged to train several DL algorithms to detect glaucomatous damage, showing a similar and better performance than experts. It is important to note that the DL model’s reliable and unreliable tests were used to train the networks.

Artificial intelligence techniques combined with the screening process and the transmission of retinal images and clinical data (teleophthalmology) demonstrate the potential to assist the specialist in making a correct diagnosis in less time, given similar (or better) results. They are achieved by analyzing DL technologies.

Teleophthalmology platforms allow the storing and forwarding of images and data in real time to screening centers. They have been improved by image processing techniques using DL techniques, increasing the offer of diagnoses at lower costs compared to in-person examinations, qualifying the diagnosis, helping reduce waiting lists for ophthalmological care, and facilitating the glaucoma screening process in more distant populations. However, future studies may establish diagnostic limits carried out through automated methodologies.

**Figure 1:**
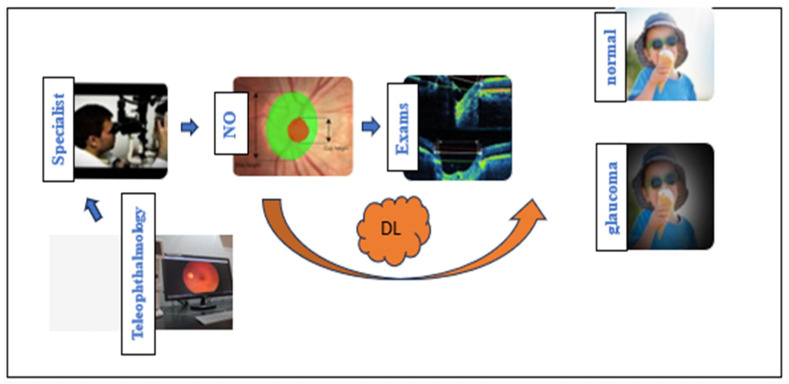
NO = Optic nerve; DL = Deep Learning. Specialists carried out glaucoma screening and DL made stages of contribution. The blue path represents the usual path for glaucoma screening and the orange arrow indicates the steps of the DL methodologies.

Figure 1 shows the steps that specialists use in glaucoma screening (blue flow), including analysis of the optic nerve head, complementary exams, and transmitting images and data via teleophthalmology. The orange flow exemplifies the applications of DL in the study of pictures of the optic nerve, VF, sdOCT, teleophthalmology, and complementary exams. The convergence of DL methodologies used in each stage of automated glaucoma screening brings more robust results closer to reality.

## 5. Conclusions

The world faces considerable challenges in eye care, including inequalities in the coverage and quality of prevention, treatment, and rehabilitation services; shortage of trained eye care providers; and weak integration of ophthalmological services into health systems, among others. In this aspect, tools such as AI have developed in recent years and can assist medical specialists in the growing population demand. It is recommended that AI-based methodologies be reflected in medical practice due to the complexity of glaucoma screening.

In the literature, DL methodologies are applied individually or jointly in several stages of glaucoma screening, including analysis of photographs of the retina, visual field, and optical coherence tomography, together with the patient’s clinical data, bringing automated screening using DL methodologies closer to manual screening carried out by specialists, improving results (mainly in suspected and early cases), facilitating access to the population, lower costs compared to face-to-face examinations, with the potential to avoid the bias of specialists in overestimating physiologically increased excavations in larger optic discs or underestimate them in small discs, in addition to facilitating the glaucoma screening process in more distant locations through teleophthalmology. However, it is essential to remember that the glaucoma diagnosis must be monitored by a qualified ophthalmologist who can recommend specific tests to confirm the diagnosis.

## Figures and Tables

**Table 1:** Example of optic nerve classification.

	Healthy ONH	Suspicious ONH		GON
Defects in the neural layer	−	+	−	+
VF	−	−	+	+
sdOCT	−	−	+	+
Conduct	Annual assessment	Complementary exams		Clinical or surgical treatment

ONH = optic nerve head, VF = visual field, sdOCT = spectral domain optical coherence tomography, GON = optic nerve with features of glaucoma, (−) absence of abnormality, (+) presence of abnormality.

**Table 2:** Example of references between ONH and GON.

	CDR	Asymmetry	Diffuse Excavation	vCDR > 0.6	Violation of ISNT Rule
Healthy ONH	CDR < 0.4 *	CDR < 0.2 *		3%	5%
GON	vCDR > 0.6	vCDR > 0.2	44%	92%	84%
Suspicious ONH		CDR > 0.2		70%	87%

* in most of the population.

**Table 3:** Main characteristics of the glaucoma screening stages.

	Costs	Portability	Interpretation/Diagnosis	Reproducibility
Images	Low	Yes, through smartphone cameras	It depends on the experience of the examiner. Does not show neuroretinal rim structures. It is a good technique for studying the progression of GON.	Dependent on location, camera, and population
VF	Moderate	Yes, VF via iPad not yet validated	The gold standard for diagnosis and treatment. It depends on the patient’s cooperation and the number of fibers involved in the structural damage.	The exam is very dependent on patient-related failures.
OCT	High	No	It is indicated in suspected cases and pre-perimetric glaucoma. It has good reproducibility but difficult interpretation and several limitations.	Good

**Table 4:** Application of DL in glaucoma screening.

Author	Objective	CE	FCF	DC	VF	OCT	E	T	R	Results
Neto [53]	Screen for glaucoma using DL segmentation and classification methods.	Images from the RIM-ONE r3, DRISHTI-GS, and REFUGE databases	yes							Disk segmentation IoU 0.94, Dice 0.97 Cup: IOU 0.81, DICE 0.96 with InceptionResNet V2;
Shuldiner [48]	Studies the risk of future progression of glaucoma based on initial VF.	ML algorithms were trained based on reliability metrics of initial VF and age.			yes				yes	ROC 0.72 [95% CI 0.70–0.75])
Kucur [49]	Using CNNs to discriminate between normal and early glaucomatous eyes.	VF of the European population, white with normal and pre-perimetric eyes.			yes				yes	Precision: Rotterdam 87.4%, Budapest 98.6%
Huang [50]	Assess VF loss using a DL system compared to ophthalmologists	Diagnosed by ophthalmologists. Use VF with false-negative and false-positive less than 30%.			yes		yes	yes	yes	Acc 85 ROC 0.93
Wen [16]	DL networks can be trained to predict VF 24-2	More than one VF 24-2 strategy and used clinical variables age, sex, and ophthalmological test.			yes				yes	PMAE = 2.47 (95% CI: 2.45 dB to 2.48 dB)
Kihara [15]	DL Networks to Predict VF from FCF, OCT	VF 24-2, combined with an sdOCT scan (exclude eyes with VF defects) caused by neurological damage and retinal disease	yes		yes	yes			yes	PMAE = 0.485 (0.438, 0.533) decibels (dB) compared with the OD; 0.060 (0.047, 0.073) dB with OCT.
Eslami [51]	CNN and RNN models predicting VF changes over time and the models’ abilities to predict VF loss.	VF with a false-positive, false-negative, and fixation losses were excluded from the study. Extra cage, sex, and the tested eye.		yes						The PMAE 95% for the CNN and 2.56 to 2.61 dB for the RNN.
Mariottoni [4]	Standard for defining glaucomatous optic neuropathy based on a set of OCT + VF+ ONH defects.	VF 24-2. SD-OCT excluded tests with a quality score.	yes	yes	yes	yes			yes	AUC 0.92 Se 77% Sp 95% GON 99.8% ONH 0.03%.
Noury [54]	To develop a 3D DL algorithm to detect glaucoma using sdOCT and fundus photographs in the population.	An assessment was conducted from scans in four countries, divided into healthy and glaucomatous eyes.				yes	yes		yes	AUC ranged from 0.91 to 0.99.
Ting [34]	Detection of various retinal pathologies using DL methodologies.	Race/ethnicity information collected.	yes				yes			GON: AUC 0.942, Se 96.4%, Sp 87.2%
Singh [24]	Evaluate DL models applied in OCT to evaluate glaucoma.	OCT images are divided into healthy and glaucoma.				yes			yes	95.68% accuracy using VGG16
Li [7]	Fundus photographs, evaluate a DL method to diagnose risk, future incidence, and glaucoma progression.	Patients visit ophthalmologists to ensure collections with open-angle and without glaucoma for control.	yes				yes		yes	AUROC incidence 0.90 AUROC progression 0.91
Lim [52]	It suggests a tool to assist in diagnosing glaucoma using fundus images.	Eyes with greater than usual axial length (axial myopia).	yes		yes	yes			yes	Xception—AUROC 93.9% for glaucoma detection

GON—glaucomatous optic neuropathy, sdOCT—spectral domain optical coherence tomography, VF—automated visual field, DL—deep learning; AUC—area under the receiver operating characteristic curve; Yes (present characteristic).

## Data Availability

Not applicable.
